# *Orthopoxvirus* Testing Challenges for Persons in Populations at Low Risk or Without Known Epidemiologic Link to Monkeypox — United States, 2022

**DOI:** 10.15585/mmwr.mm7136e1

**Published:** 2022-09-09

**Authors:** Faisal S. Minhaj, Julia K. Petras, Jennifer A. Brown, Anil T. Mangla, Kelly Russo, Christina Willut, Michelle Lee, Jason Beverley, Rachel Harold, Lauren Milroy, Brian Pope, Emily Gould, Cole Beeler, Jack Schneider, Heba H. Mostafa, Shana Godfred-Cato, Eleanor S. Click, Brian F. Borah, Romeo R. Galang, Shama Cash-Goldwasser, Joshua M. Wong, David W. McCormick, Patricia A. Yu, Victoria Shelus, Ann Carpenter, Sabrina Schatzman, David Lowe, Michael B. Townsend, Whitni Davidson, Nhien T. Wynn, Panayampalli S. Satheshkumar, Siobhán M. O’Connor, Kevin O’Laughlin, Agam K. Rao, Andrea M. McCollum, María E. Negrón, Christina L. Hutson, Johanna S. Salzer, CDC Monkeypox Emergency Response Team, CDC Monkeypox Emergency Response Team, Ramona Bhatia, Anne Kimball, Brett Petersen, Yon Yu, Kevin Chatham-Stephens, Kia Padgett, Maureen J. Miller, Isaac Zulu, William C. Carson, Sapna Bamrah Morris, Caroline Schrodt, Amy Beeson, David Kuhar, Zeshan Chisty

**Affiliations:** ^1^CDC Monkeypox Emergency Response Team; ^2^Epidemic Intelligence Service, CDC; ^3^Indiana Department of Health; ^4^District of Columbia Department of Health; ^5^Howard County Health Department, Columbia, Maryland; ^6^Indiana University School of Medicine, Indianapolis, Indiana; ^7^Johns Hopkins Medical Institute, Baltimore, Maryland; ^8^Laboratory Leadership Service, CDC.; CDC; CDC; CDC; CDC; CDC; CDC; CDC; CDC; CDC; CDC; CDC; CDC; CDC; CDC

Since May 2022, approximately 20,000 cases of monkeypox have been identified in the United States, part of a global outbreak occurring in approximately 90 countries and currently affecting primarily gay, bisexual, and other men who have sex with men (MSM) (*1*). *Monkeypox virus* (MPXV) spreads from person to person through close, prolonged contact; a small number of cases have occurred in populations who are not MSM (e.g., women and children), and testing is recommended for persons who meet the suspected case definition* (*1*). CDC previously developed five real-time polymerase chain reaction (PCR) assays for detection of orthopoxviruses from lesion specimens (*2*,*3*). CDC was granted 510(k) clearance for the nonvariola-orthopoxvirus (NVO)–specific PCR assay by the Food and Drug Administration. This assay was implemented within the Laboratory Response Network (LRN) in the early 2000s and became critical for early detection of MPXV and implementation of public health action in previous travel-associated cases as well as during the current outbreak (*4*–*7*). PCR assays (NVO and other *Orthopoxvirus* laboratory developed tests [LDT]) represent the primary tool for monkeypox diagnosis. These tests are highly sensitive, and cross-contamination from other MPXV specimens being processed, tested, or both alongside negative specimens can occasionally lead to false-positive results. This report describes three patients who had atypical rashes and no epidemiologic link to a monkeypox case or known risk factors; these persons received diagnoses of monkeypox based on late cycle threshold (Ct) values ≥34, which were false-positive test results. The initial diagnoses were followed by administration of antiviral treatment (i.e., tecovirimat) and JYNNEOS vaccine postexposure prophylaxis (PEP) to patients’ close contacts. After receiving subsequent testing, none of the three patients was confirmed to have monkeypox. Knowledge gained from these and other cases resulted in changes to CDC guidance. When testing for monkeypox in specimens from patients without an epidemiologic link or risk factors or who do not meet clinical criteria (or where these are unknown), laboratory scientists should reextract and retest specimens with late Ct values (based on this report, Ct ≥34 is recommended) (*8*). CDC can be consulted for complex cases including those that appear atypical or questionable cases and can perform additional viral species- and clade-specific PCR testing and antiorthopoxvirus serologic testing.

The three patients described in this report were not MSM, and all had an atypical rash (i.e., without the characteristic progression over 2–4 weeks from pustular to deep-seated, umbilicated lesions). The patients initially received positive *Orthopoxvirus* real-time PCR test results, with high Ct values (≥34); the positive PCR results were followed by implementation of clinical and public health recommendations for monkeypox, including antiviral treatment and PEP.^†^ This activity was reviewed by CDC and was conducted consistent with applicable federal law and CDC policy.^§^

## Description of Patients

Patient A, a healthy pregnant woman (estimated 37 weeks’ gestation) was evaluated for labor and was noted to have a pruritic erythematous rash on her arms, abdomen, upper back, calves, and shins. Her lesions, not typical for monkeypox, had irregular borders, and were different sizes and in different stages of development (i.e., tan papules, crusted papules, pustules, and hyperpigmented macules) in the same anatomic locations, with reported onset 5 weeks earlier. No genital lesions were present. She did not report typical prodromal signs or symptoms of monkeypox (e.g., body aches, lymphadenopathy, fever, or chills). A household member was reported to have a similar rash, with onset 4 days before that in patient A; that person’s rash resolved within 1 week, and no testing was performed; no epidemiologic link to a person with monkeypox was identified. Patient A had no interstate or international travel during the 3 weeks preceding rash onset. She reported varicella infection and receipt of smallpox vaccination as a child. Tests for varicella-zoster virus, syphilis, herpes simplex virus, cryptococcosis, and histoplasmosis were performed, all with negative results. A swab from a pustular forearm lesion, obtained 53 days after rash onset yielded a positive NVO test result (Table). Two days after receiving the result, the woman had an uncomplicated vaginal delivery of a healthy neonate. The state health department and CDC clinicians recommended several measures until lesions resolved: 1) initiation of monkeypox infection-control precautions^¶^ in the hospital, 2) precautions to prevent skin-to-skin contact between mother and infant,** 3) designation of another household member as the primary caregiver, 4) delay of breastfeeding, and 5) disposal of breast milk. Because of concern for congenital or perinatal transmission, vaccinia immune globulin intravenous (VIGIV) was administered to the neonate under a single patient emergency Investigational New Drug application. Further testing with a Clade II (i.e., West African) MPXV–specific real-time PCR LDT was inconclusive. Because of the discordant results, serum from patient A obtained on day 42 after rash onset was sent to CDC for serologic analysis; no antiorthopoxvirus antibodies were detected, arguing against orthopoxvirus infection (*9*). The recommendations restricting contact with the baby and for delaying breastfeeding were discontinued after rash resolution when the infant was aged 21 days (Figure). The patient’s skin lesions were most likely attributable to bed bugs, which was a diagnosis that the clinical care team considered initially but set aside upon receipt of the positive NVO result.

**TABLE:** Characteristic of and testing, interventions, and treatment given to persons initially receiving monkeypox diagnoses based on a false-positive test result— United States, 2022

Patient	Patient characteristic	Symptoms	Initial real-time PCR test result*	Additional MPXV, NVO, or OPXV real-time PCR test result*	IgM^†^	Treatment administered	Total no. of contacts who received PEP^§^ (adults, children)	Suspected alternative diagnosis
A	Pregnant woman, 37 weeks’ gestation	Rash, pruritus	**Pos**	MPXV: inconclusive^¶^	Neg	Tecovirimat to patient A, VIGIV to neonate	1 (1, 0)	Bed bugs
			NVO Ct: 34.30					
B	Elementary school-aged child	Rash, fatigue, headache, decreased appetite, fever	**Pos**	Neg	NP	Tecovirimat	4 (2, 2)	Hand, foot, and mouth disease
			NVO Ct: 35.82	NVO Ct: >40**				
C	Infant	Diarrhea, lymphadenopathy, fever, rash	**Pos**	MPXV: Inconclusive^¶^	Neg	Tecovirimat	19 (12, 7)	Pending
			NVO Ct: 34.67	Neg				
			OPVX Ct: 36.71	OPXV Ct: >40				
				Neg				
				NVO Ct >40				

**Abbreviations**: Ct = cycle threshold; IgM = immunoglobulin M; MPXV = *Monkeypox virus*; Neg = negative; NP = not performed; NVO = non-variola *Orthopoxvirus*; OPXV = *Orthopoxvirus*; PCR = polymerase chain reaction; PEP = postexposure prophylaxis; VIGIV = vaccinia immune globulin intravenous.

* Real-time PCR assays for testing of orthopoxviruses and *Monkeypox virus*–specific assays have varying Ct cutoffs depending on assay used. Cutoffs can range from approximately 37 to 40.

^†^ Antiorthopoxvirus IgM antibody is expected to be detectable 4–56 days after rash onset in patients with monkeypox.

^§^ JYNNEOS vaccine.

^¶^ Test results from duplicate swab from the initial lesion, inconclusive based on internal control indicating inadequate specimen collection.

** Test results from a reextraction and retesting of the initial lesion swab.

**FIGURE:**
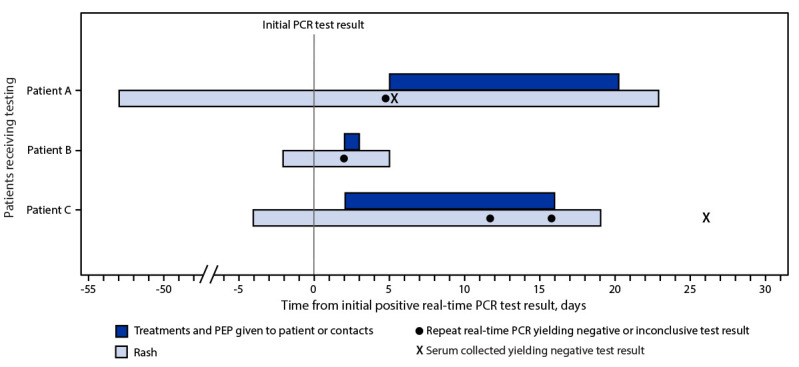
Timeline of patient testing and public health interventions for false-positive *Monkeypox virus* test results — United States, 2022 **Abbreviations:** PCR = polymerase chain reaction; PEP = postexposure prophylaxis.

Patient B is an elementary school–aged, previously healthy child (Table). The child developed influenza-like symptoms followed 2 days later by raised lesions on the face. The next day, lesions had spread to the trunk, back, and arms. The lesions were initially papulopustular, and over the course of 2 days became ulcerated and crusted. No epidemiologic link to a person with monkeypox was identified. A swab of a facial lesion tested positive by an orthopoxvirus generic LDT. Treatment with tecovirimat was started because the child had periorbital lesions and because of concern for potential ocular autoinoculation and development of sight-threatening disease. The child lived with four other persons and had engaged in a contact sport when the rash was present. The child isolated at home, and all family members received PEP with JYNNEOS vaccine; PEP for teammates was held pending reextraction and retesting of the original specimen (Figure). The subsequent result was negative, and the child was released from isolation. Enterovirus PCR testing was positive, suggesting a diagnosis of hand, foot, and mouth disease.

Patient C is an infant who visited the United States with both parents for approximately 1 month and subsequently traveled to another country with four other families for vacation. During that trip, the infant experienced diarrhea followed by lymphadenopathy, and 2 days later, after returning to the United States, developed fever and a rash (Table). The rash was described as maculopapular and vesicular, and started on the arms and legs progressing to the earlobe, chest, scalp, and lower abdomen; the rash scabbed over 2 weeks later. One abdominal lesion tested positive by NVO and an orthopoxvirus generic LDT; two other lesions tested negative. The infant was treated with oral tecovirimat. No epidemiologic link to a person with monkeypox was identified. Over a 15-day period starting on the second day of the vacation, five of 11 children (including patient C) and four of 14 adults from the families who vacationed with the infant experienced rashes that varied in appearance. Among some of the children, the rash looked like insect bites and not consistent with monkeypox; among others, the rash was vesicular or pustular involving the arms, legs, feet, fingers, or face, and eventually scabbing over. Results of NVO testing of lesions on four children and four adults were negative or inconclusive. A multijurisdictional investigation was launched to determine potential exposures and administer PEP to all family members. Twelve adults and seven children (aged 0–14 years) received PEP with JYNNEOS. Because of the ongoing investigation, multiple families changed travel plans, and patient C’s family postponed travel back to their country of residence for approximately 4 weeks. Serum from two adults and four children (including patient C) obtained 3–31 days after rash onset did not detect the presence of antiorthopoxvirus antibodies (Figure).

## Discussion

Evaluation of these three patients for monkeypox highlights the need for caution in interpreting single laboratory test findings in patients with a low pretest probability of infection; this includes lack of an epidemiologic link, non-MSM populations (e.g., women and children, who currently account for <2% of confirmed monkeypox cases), and signs, symptoms, or rash progression inconsistent with monkeypox. This approach is similar to the caution recommended in evaluating other laboratory tests when pretest probability is low (e.g., D-dimer results for a deep vein thrombosis or serology for Lyme disease)^††^ (*10*). Multiple clinical features in each of these three patients were inconsistent with monkeypox, including an atypical rash that was inconsistent with the characteristic progression of monkeypox lesions, as well as the absence of an epidemiologic link to a known case of monkeypox. The Ct values of all initial positive test results were high (≥34) indicating a low level of viral DNA. Cautious interpretation of test results is warranted when the pretest probability of monkeypox is low. As monkeypox testing has expanded, CDC recommends that laboratory professionals verify positive diagnostic results (*8*) for *Orthopoxvirus* or MPXV DNA in specimens with high Ct values, especially from persons who do not meet epidemiologic risk criteria for monkeypox or for whom lesions do not progress as expected. Molecular tests (e.g., real-time PCR tests) are highly specific and sensitive; however, when epidemiologic criteria are absent or unknown and the Ct value is high (generally ≥34), CDC recommends reextraction and retesting of the specimen.

Monkeypox currently occurs predominantly among MSM, although infection can occur in any person after close physical contact with persons with monkeypox or items that have been in contact with lesions, such as clothing or bedding. Because the positive predictive value in populations with low disease incidence is lower than that in populations with a higher disease incidence, laboratory results in persons with low pretest probability of infection should be carefully examined and reviewed, and other plausible diagnoses (e.g., hand, foot, and mouth disease; varicella; molluscum contagiosum) should be considered. The clinical course of illness should be reviewed, including documenting the lesions with photographs. CDC can be consulted for atypical or questionable cases and can perform additional viral-specific and clade-specific PCR testing and antiorthopoxvirus serology.

**Summary**

**What is already known about this topic?**

Testing for *Monkeypox virus*, using Food and Drug Administration 510(k)–cleared non-variola *Orthopoxvirus* real-time polymerase chain reaction (PCR) test and laboratory developed real-time PCR tests, is critical for diagnosis of suspected cases.

**What is added by this report?**

Three persons with atypical rashes, uncharacteristic illnesses, and absence of risk factors or an epidemiologic link to a known monkeypox case received false-positive real-time PCR test results; late cycle threshold values were all ≥34.

**What are the implications for public health practice?**

When testing specimens from patients with atypical signs and symptoms or without epidemiologic links or risk factors or where these are unknown, laboratories should reextract and retest specimens with real-time PCR Ct values that are high (≥34) to avoid unnecessary medical treatment and expenditure of public health resources.

## Footnotes

*
https://www.cdc.gov/poxvirus/monkeypox/clinicians/case-definition.html

†
https://www.cdc.gov/poxvirus/monkeypox/health-departments/vaccine-considerations.html

§ 45 C.F.R. part 46, 21 C.F.R. part 56; 42 U.S.C. Sect. 241(d); 5 U.S.C. Sect. 552a; 44 U.S.C. Sect. 3501 et seq.

¶
https://www.cdc.gov/poxvirus/monkeypox/clinicians/infection-control-healthcare.html

**
https://www.cdc.gov/poxvirus/monkeypox/clinicians/pregnancy.html

††
https://www.cdc.gov/lyme/resources/pdfs/lyme-1532_poster_prior-pretest-probability-testing_digital-508.pdf
